# Multiregional origins of the domesticated tetraploid wheats

**DOI:** 10.1371/journal.pone.0227148

**Published:** 2020-01-22

**Authors:** Hugo R. Oliveira, Lauren Jacocks, Beata I. Czajkowska, Sandra L. Kennedy, Terence A. Brown

**Affiliations:** School of Earth and Environmental Sciences, Manchester Institute of Biotechnology, University of Manchester, Manchester, England, United Kingdom; Institute of Genetics and Developmental Biology Chinese Academy of Sciences, CHINA

## Abstract

We used genotyping-by-sequencing (GBS) to investigate the evolutionary history of domesticated tetraploid wheats. With a panel of 189 wild and domesticated wheats, we identified 1,172,469 single nucleotide polymorphisms (SNPs) with a read depth ≥3. Principal component analyses (PCAs) separated the *Triticum turgidum* and *Triticum timopheevii* accessions, as well as wild *T*. *turgidum* from the domesticated emmers and the naked wheats, showing that SNP typing by GBS is capable of providing robust information on the genetic relationships between wheat species and subspecies. The PCAs and a neighbour-joining analysis suggested that domesticated tetraploid wheats have closest affinity with wild emmers from the northern Fertile Crescent, consistent with the results of previous genetic studies on the origins of domesticated wheat. However, a more detailed examination of admixture and allele sharing between domesticates and different wild populations, along with genome-wide association studies (GWAS), showed that the domesticated tetraploid wheats have also received a substantial genetic input from wild emmers from the southern Levant. Taking account of archaeological evidence that tetraploid wheats were first cultivated in the southern Levant, we suggest that a pre-domesticated crop spread from this region to southeast Turkey and became mixed with a wild emmer population from the northern Fertile Crescent. Fixation of the domestication traits in this mixed population would account for the allele sharing and GWAS results that we report. We also propose that feralization of the component of the pre-domesticated population that did not acquire domestication traits has resulted in the modern wild population from southeast Turkey displaying features of both the domesticates and wild emmer from the southern Levant, and hence appearing to be the sole progenitor of domesticated tetraploids when the phylogenetic relationships are studied by methods that assume a treelike pattern of evolution.

## Introduction

Tetraploid emmer wheat (*Triticum turgidum* L. subsp. *dicoccum* [Schrank ex Schübl.] Thell.), the cultivated form of *T*. *turgidum* L. subsp. *dicoccoides* (Korn. ex Asch. & Graebn.) Thell., was among the first plant species to be domesticated in the Fertile Crescent of southwest Asia [1]. Emmer has hulled grain, but from it evolved the naked tetraploid wheats such as durum (*T*. *turgidum* L. subsp. *durum* [Desf.] Husn.), rivet wheat (*T*. *turgidum* L. subsp. *turgidum* (Desf.] Husn.) and the *turanicum*, *polonicum* and *carthlicum* subspecies. All of the naked wheats are fully domesticated but they have different ear characteristics and environmental requirements. It is not known if they emerged independently from domesticated emmer or if there was a common naked wheat ancestor: some molecular studies indicate genetic uniformity between different naked subspecies and other studies report regional or taxonomic differences [2–4]. In a separate series of evolutionary events [1,5], emmer hybridized with the wild goatgrass *Aegilops tauschii* Coss. to give a hexaploid lineage that includes bread wheat, *Triticum aestivum* L., the foremost crop of modern agriculture [6]. The domestication of wild emmer was therefore a critical stage in the transition from a hunting-gathering mode of subsistence to one based on agriculture.

All members of the *T*. *turgidum* species have A^u^A^u^BB genomes (2n = 4x = 28 chromosomes) and are annual, predominantly self-pollinated plants [1]. Wild emmer today is an ecological specialist found mainly on basaltic and limestone bedrocks in the upper Jordan Valley with patchy distribution in southeast Turkey, northwest Syria and in the mountainous regions of eastern Iraq/western Iran [7,8], the stands in the Jordan Valley displaying the greatest morphological and genetic diversity [8]. Emmer is distinct from a second type of cultivated tetraploid wheat, *T*. *timopheevii* (Zhuk.) Zhuk. subsp. *timopheevii*, which is the domesticated form of *T*. *timopheevii* (Zhuk.) Zhuk. subsp. *armeniacum* (Jakubz.) Slageren. The *T*. *timopheevii* wheats have A^m^A^m^GG genomes (2n = 4x = 28) and can form hybrids with *T*. *turgidum*, but these hybrids are sterile due to extensive chromosomal irregularities that occur during meiosis [9]. Domesticated *T*. *timopheevii* is mainly found in western Georgia and its wild progenitor occurs only in southeast Turkey, north Syria, the Iraq/Iran border and Transcaucasia where it is sympatric with wild emmer [10]. *Triticum timopheevii* seems to have always been a secondary crop, although it is unclear why *T*. *turgidum* species were preferred by early farmers [1].

The archaeological data suggest that the relatively dense stands of wild emmer in the Jordan Valley were the source of the first cultivated plants. The presence of extensive assemblages of wild emmer at southern Levant sites such as Ohalo II and Netiv HaGdud suggests that the plant was intensively collected by people of the local Natufian culture (c.13,000–9,500 BC) and was very likely cultivated in this region during the Pre-Pottery Neolithic A (PPNA, c.9,500–8,500 BC) [11–14]. Emmer is absent at contemporaneous sites in the upper Euphrates and southeast Turkey, such as Abu Hureyra, Göbekli, Cayönü, where wild einkorn and barley were cultivated [12,15]. During the subsequent Pre-Pottery Neolithic B (PPNB, c.8,500–6,500 BC), domesticated emmer appears throughout the Fertile Crescent, the earliest spikelets with the rough abscission scar characteristic of a non-shattering seed head, which is looked on as a key domestication trait, being found at Tell Qarassa in southern Syria by 8700–8200 BC [13,14] and Cayönü and Cafer Höyük in southeast Turkey at 8,250–7,550 BC [1]. Based on grain size and shape, which some archaeobotanists believe can also be used as diagnostic features for domesticated cereals [16], there is additional evidence for mixed cultivation of wild and domesticated emmer for almost a millennium during the PPNB at Tell Aswad and Jericho in the southern Levant [11], accompanied by diffusion of wild emmer to Cyprus around 8,650–7,550 BC [1]. At about 7,800 BC, fully domesticated emmer appears in increasing amounts in Chogha Golan in the Zagros mountains, apparently introduced from elsewhere [17,18]. The naked wheats emerged shortly after emmer, first appearing in the archaeological record during the 8^th^ millennium BC at Tell Aswad and Aşikli Höyük, the latter in Turkey. Although it is difficult to distinguish hexaploid and tetraploid wheats in archaeological remains, most archaeobotanists agree that these early forms must have been tetraploid [1]. Being part of the earliest package of crops, they quickly replaced hulled wheats in many regions [19].

The emphasis that the archaeological record places on the southern Levant as the origin of emmer cultivation was not supported by the first genetic studies of domesticated tetraploid wheats. Analysis of amplified fragment length polymorphisms (AFLPs) in wild emmer accessions and in cultivated emmer and durum lines initially identified a single localized origin for emmer domestication in the Karaca Dağ mountains, to the southwest of Diyarbakir in southeast Turkey [20]. This conclusion agreed with a previous study of einkorn, also based on AFLP analysis, which also identified the Karaca Dağ region as a domestication centre [21]. However, an investigation of chloroplast microsatellite haplotypes suggested that there were at least two independent domestication events, one of which was proposed to have taken place in the Kartal Daği region, adjacent to Gaziantep, some 280 km west of Karaca Dağ [22]. An extension of the AFLP analysis to include additional wild accessions failed to confirm the Kartal Daği as a site of emmer domestication, identifying the closest wild relatives of domesticated emmer in the Karaca Dağ and in the Sulaymaniyah region of Iran and Iraq [23]. Restriction fragment polymorphisms (RFLP) analysis then confirmed the genetic distinction between the southern and northern wild populations and placed the domesticates closest to northern wild emmer from the Diyarbakir region, though with significant affinity also with the wild population in the southern Levant [24]. The authors interpreted these results as indicating that emmer was either domesticated independently in the northern and southern parts of the Fertile Crescent, or that domestication occurred in the Diyarbakir region and was followed by gene flow into the crop from wild populations in the southern Levant. The latter hypothesis was later supported by a reanalysis of the original AFLP data [12], but a more recent study of the *Brittle rachis* genes controlling ear shattering, a key domestication trait, has again indicated that the southern Levant played an important role in emmer domestication [25].

We have previously suggested that the contradiction between the southern origin for emmer cultivation as supported by the archaeological data and the northern origin indicated by genetics might be due to the role of gene flow being greater than suspected [26]. If this is the case, then the true relationships between the crop and its wild progenitors would not be revealed by phylogenetic methods that assume a treelike pattern of evolution [27,28]. This is because the relationship will be reticulate rather than treelike, introgression leading to different parts of the domesticated genome having different genealogical histories. Taking this possibility into account, we constructed supernetworks from nuclear gene sequences and retrotransposon insertion data and found a complex reticulate evolutionary relationship between wild emmer and the hulled and naked domesticates, suggesting extensive hybridization between the wild and cultivated populations [28], consistent with reports of the importance of gene flow during the evolution of other crops such as barley [29,30] and rice [31].

In recent years, the development of genotyping-by-sequencing (GBS), coupled with complexity reduction methods such as reduced-representation sequencing, has enabled the rapid scoring of hundreds of thousands of single nucleotide polymorphisms (SNPs) in multiple versions of a single genome [32,33]. The resulting data can provide much more detailed information on the diversity of different parts of a genome than has previously been possible by analysis of RFLPs and AFLPs, and hence enable the evolutionary histories of different loci to be examined and compared with greater precision. Here we report the use of GBS to investigate the origins and evolutionary history of the domesticated tetraploid wheats.

## Materials and methods

### Wheat accessions

The study material consisted of 189 tetraploid wheat accessions (S1 Table) which, according to the germplasm identifications, comprised eleven *Triticum timopheevii* subsp. *armeniacum*, eight *T*. *timopheevii* subsp. *timopheevii*, 76 wild emmer (*T*. *turgidum* subsp. *dicoccoides*), 42 domesticated emmer (*T*. *turgidum* subsp. *dicoccum*) including one described as *T*. *ispahanicum* Heslot (a hulled domesticated wheat sometimes considered distinct from emmer; [34]) and 52 naked tetraploid wheats (27 durum wheats [*T*. *turgidum* subsp. *durum*], eleven rivet wheats [*T*. *turgidum* subsp. *turgidum*], six Khorasan wheats [*T*. *turgidum* L. subsp. *turanicum* (Jakubz) Á. & D. Löve)], five Polish wheats [*T*. *turgidum* L. subsp. *polonicum* (L.) Thell.] and three Persian wheats [*T*. *turgidum* L. subsp. *carthlicum* (Nevski) Á. & D. Löve]).

During the early analysis, fourteen of these accessions were reclassified (see Results): eleven accessions identified by the germplasm collection as wild emmer were reclassified as *T*. *timopheevii* subsp. *armeniacum*; one described as *T*. *timopheevii* subsp. *armeniacum* was reclassified as wild emmer; and one *T*. *timopheevii* subsp. *timopheevii* as well as one of the Khorasan wheat accessions were classified as domesticated emmer. The final accession set therefore comprised 21 *Triticum timopheevii* subsp. *armeniacum*, seven *T*. *timopheevii* subsp. *timopheevii*, 66 wild emmer, 44 domesticated emmer, and 51 naked wheats (27 durum, eleven rivet, five Khorasan, five Polish and three Persian). These reclassifications are listed in S1 Table.

Accessions were selected to cover the full extent of the geographical distribution for each of the taxa. In particular the 66 wild emmers included 35 accessions collected from the southern Levant (referred to as ‘south’ or ‘S’ in Results), eleven from the Karaca Dağ region (KD), 13 from other northern parts of the Fertile Crescent (N) with one outlier in Armenia, and seven accessions from the eastern Fertile Crescent (E) with outliers whose collection sites are described as Tehran (S1 Fig, S1 Table).

### DNA extraction and genotyping-by-sequencing

Seeds were vernalized for 2 days at 4°C and grown for two weeks at room temperature in petri dishes covered with filter paper. DNA was extracted from first leaves using the Bioline Isolate II Plant DNA Kit. In a few cases where germination did not occur DNA was extracted from pulverized seeds with the Roche HighPure PCR Product Purification Kit. DNA was quantified by a Qubit dsDNA HS assay with a Qubit 2.0 Fluorometer and DNA integrity was checked by electrophoresis in 1% agarose gels. The resulting samples had DNA concentrations between 30–100 ng μl^–1^.

Genotyping-by-sequencing (Genomic Diversity Facility, Cornell University) was carried out as described by Elshire et al. [35]. Optimization was attempted with *Pst*I and *Eco*T221 and the former chosen for genome complexity reduction. Unique sequence tags were identified from the FASTQ files and aligned to release 31 of the *T*. *aestivum* genome using BWA v.0.7.8-r455 [36]. SNP calling was carried out with the TASSEL-GBS pipeline [37] and the *vcf* files were handled with VCFtools v.0.1.13 [38] and TASSEL [39]. Different filters for coverage, missing data, biallelic SNPs, indels and minimum allele frequency were applied as described in Results. The GBS data are available at the European Nucleotide Archive, study PRJEB42105.

### Data analysis

Allele frequencies and genetic diversity measures were calculated using VCFtools and TASSEL, pairwise *F*_ST_ values were calculated using VCFtools, genetic distance and kinship matrixes were computed using TASSEL and visualised with the R gplots package [40], and principal components analysis (PCA) was performed with TASSEL. An unrooted neighbour-joining (NJ) tree was produced in TASSEL. Population structure was examined with the model-based clustering algorithm *STRUCTURE 2*.*3*.*4* [41], with *K* values between 1 and 12, 20,000 burnins, 40,000 MCMC repetitions and ten independent runs for each value of *K*. *Q*-matrixes were displayed on geographical maps using ArcMap v.10 of ArcGIS [42]. Graphs were plotted in Excel and histograms constructed with XLSTAT 2018.

A genome-wide association study (GWAS) was performed to identify SNPs associated with the domesticated phenotype, using a mixed linear model with the first five components of a PCA as input. A kinship matrix (*Q*+*K* model) was computed using TASSEL, and a Bonferroni correction with α = 0.01 was used to highlight significant marker-trait associations [43]. Manhattan plots were obtained with the R package *qqman* [44].

## Results

### Genotyping-by-sequencing

Complexity reduction with *Pst*I was found to be suitable for tetraploid wheat DNA. Sequencing of the 189 accessions yielded a total of 605 million reads from which 6,962,191 tags with a minimum of three reads per tag were obtained. Of these, 40.3% mapped to unique positions in the *T*. *aestivum* reference genome, 48.1% mapped to multiple positions and 11.7% were unmapped. From the uniquely mapped tags 1,172,469 SNPs were extracted with an average read depth of 3.5× and an average of 48.95% missing data (S2 Table). The amount of missing data is high but this is not uncommon with GBS [45]. Three of the 189 accessions (the northern wild emmer IG 109085 and two rivet wheats, PI 94689 and PI 166496; S1 Table) were not studied further as these had >90% missing data.

Among the other 186 accessions, the proportion of heterozygous sites was 7.46%, the average minor allele frequency was 0.133, and the SNPs comprised 53.8% transitions, 36.8% transversions and 9.4% indels. The majority of SNPs mapped to the A and B genomes (44.3 and 49.0%, respectively) but 6.0% mapped to the D genome with the remainder (0.7%) mapping to contigs without chromosome information (S3 Table). The apparent presence of D genome SNPs suggests errors in the mapping pipeline or in the genome assembly quality, considering that the samples included only tetraploid wheats with A^u^B genomes (*T*. *turgidum*) or A^m^G genomes (*T*. *timopheevii*). We assume that G genome SNPs from the *T*. *timopheevii* accessions mapped to the B genome of the *T*. *aestivum* reference sequence, in view of the close similarity between these two genomes [46]. Reliable mapping is indicated by the fact that for the A and B genomes, but not the D genome, the number of SNPs mapped per chromosome is roughly proportional to the size of each chromosome (S2 Fig).

The proportion of missing data and the number of sites without missing data varied among the taxa, being highest with *T*. *timopheevii* subsp. *armeniacum* and *T*. *timopheevii* subsp. *timopheevii* (S4 Table). As well as possible stochastic differences in DNA quality and library preparation, the higher amount of missing data with the A^m^G wheats might reflect evolutionary differences compared with the *T*. *aestivum* reference genome. Genetic diversity calculated as π [47] was higher for the wild taxa (*T*. *timopheevii* subsp. *armeniacum* and *T*. *turgidum* subsp. *dicoccoides*) compared with their domesticated subspecies (S4 Table). Among the wild emmers, the south accessions had higher diversity than any of the other geographical groups. The latter observation remained true even when the north, Karaca Dağ and east wild emmers were placed in a single group. Tajima’s D [48] was negative for the *T*. *turgidum* subspecies, indicating recent population expansion or selective sweeps.

### Population structure

**Fig 1:**
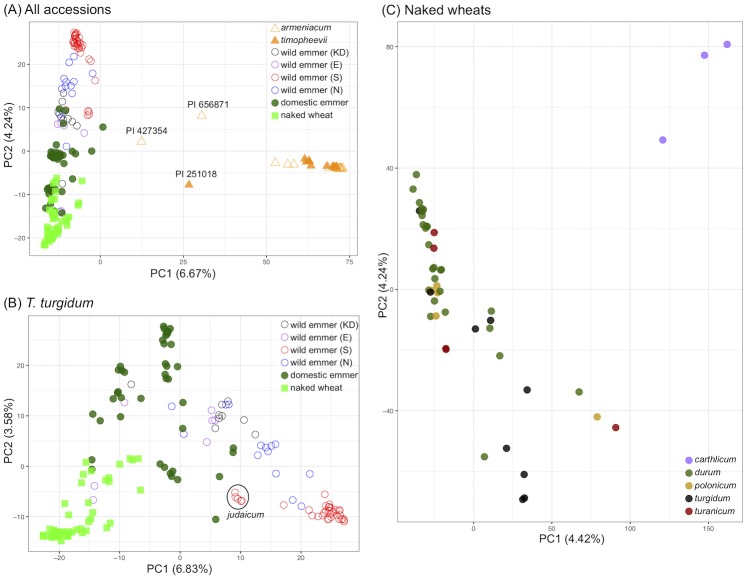
PCAs based on a filtered subset of 51,365 SNPs (no indels, minor allele frequency ≥0.05, minimum depth coverage 5×, <20% missing data). (A) 186 *T*. *turgidum* and *T*. *timopheevii* accessions, (B) 158 *T*. *turgidum* accessions, and (C) 49 naked wheat accessions. In panel A the three accessions occupying intermediate positions between the *T*. *turgidum* and *T*. *timopheevii* clusters are labelled, and in panel B the cluster of accessions including two identified as members of the *judaicum* race is circled. To avoid confusion, the symbols in panels A and B give the taxonomic identification of each accession after the reclassifications described in the text.

PCAs were initially performed on 51,365 SNPs that remained after filtering for no indels, minor allele frequency ≥0.05, minimum depth coverage of 5× and no more than 20% missing data. When all 186 accessions were included, the first PC separated the *T*. *turgidum* and *T*. *timopheevii* accessions (Fig 1A). Given the different evolutionary lineages of *T*. *turgidum* and *T*. *timopheevii* the separation between the groups is expected and demonstrates the validity of GBS SNP analysis as a way of classifying tetraploid wheat accessions. Several accessions did, however, occupy anomalous positions in the PCA. Ten accessions described by the germplasm collections as *T*. *turgidum* subsp. *dicoccoides* (PI 560697, PI 560873, PI 560877, PI 656869, PI 656872, PI 656873, CGN16098, CGN16102, CGN13161 and CGN24296) clustered with the *T*. *timopheevii* subsp. *armeniacum* accessions, and one described as *T*. *timopheevii* subsp. *armeniacum* (PI 427998) clustered with *T*. *turgidum* subsp. *dicoccoides*. Sequencing of the *Ppd-B1* and *Ppd-G1* genes confirmed that each of these eleven accessions had been misclassified by the germplasm curators [49]. Additionally, one sample described as *T*. *timopheevii* subsp. *timopheevii* (PI 190974) clustered with *T*. *turgidum* subsp. *dicoccum*. *Ppd-1* typing indicated that the seed from which DNA was obtained was *T*. *turgidum* subsp. *dicoccum*, although a second seed from PI 190974 appeared to be *T*. *timopheevii*. For the purposes of this project, this accession was reclassified as a cultivated emmer. One accession, PI 113393, which was described as *T*. *turgidum* subsp. *turanicum*, also clustered with *T*. *turgidum* subsp. *dicoccum*, and was reclassified as cultivated emmer. The reclassifications of these fourteen accessions are given in S1 Table and were used in subsequent analyses.

Two additional *T*. *timopheevii* accessions (PI 251018 and PI 427354) and one described as *T*. *turgidum* subsp. *dicoccoides* (PI 656871) occupied intermediate positions between the *T*. *turgidum* and *T*. *timopheevii* clusters (indicated on Fig 1A). Further typing of PI 656871 suggested that this accession was not *T*. *turgidum*, and it was reclassified as *T*. *timopheevii* subsp. *armeniacum* although its exact status remained unclear (S5 Table). The taxonomies of PI 251018 and PI 427354 were also uncertain, PI 251018 appearing to be a nuclear hybrid with *T*. *timopheevii* cytoplasm, and PI 427354 possibly being an example of *Triticum zhukovskyi* Menabde & Ericzjan, a wild hexaploid wheat with A^m^A^m^A^m^A^m^GG genomes (see Discussion). For these two accessions, the germplasm identifications as *T*. *timopheevii* were retained.

The PCA of the 186 accessions also separated, on the second axis, the wild emmers from the domesticated emmers and naked wheats. This separation was clearer when the PCA was carried out with just these 158 *T*. *turgidum* accessions (Fig 1B). This PCA also shows clustering of the naked wheats and domesticated emmers into separate groups, as well as separation between the wild emmers from the southern Levant and a mixed group comprising the other wild emmers (i.e. those from the northern Fertile Crescent, the Karaca Dağ region and the eastern Fertile Crescent). The south wild emmers include two members (PI 414721, PI 538680) previously identified as members of the *judaicum* race [12], which has distinct morphological and karyotypic features [50,51]. These two accessions cluster in a distinct position in the PCA (circled in Fig 1B), along with PI 414718 and PI 467004, whose collection sites were located within the range of *judaicum* on the northwest shore of the Sea of Galilee, and PI 538705, collected from a site some 50 km further north in Lebanon. A third PCA was carried out with the 49 naked wheat accessions (Fig 1C). Both the first and second PCs separated the three *T*. *turgidum* subsp. *carthlicum* accessions from the other naked wheats, the latter group displaying no clear separation between the subsp. *durum*, *turgidum*, *turanicum* and *polonicum* accessions.

To assess if parameters used for SNP filtering affected the inferred population structure, PCAs for the 186 accessions were also produced with different degrees of coverage (no filtering, ≥5× coverage, ≥10× coverage) and missing data (no filtering, ≤20% missing data, and zero missing data). The same clustering pattern was observed for all combinations of parameters (S3 Fig).

**Fig 2:**
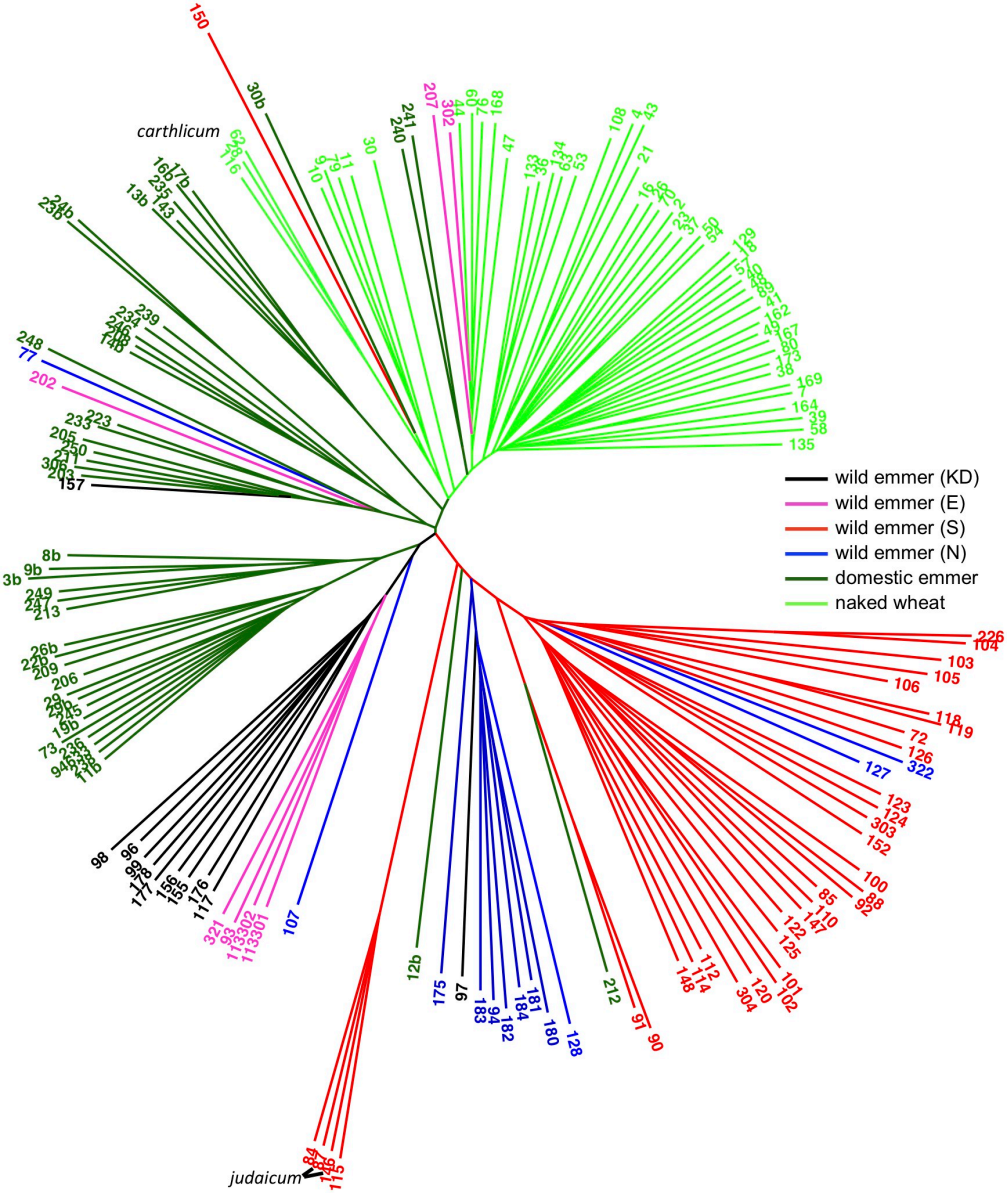
Neighbour joining tree for 158 *T*. *turgidum* accessions based on 1,172,469 SNPs. The positions of the three *T*. *turgidum* subsp. *carthlicum* accessions and the two members of the *judaicum* race of *T*. *turgidum* subsp. *dicoccoides* are marked. Branches are labelled with code numbers as listed in S1 Table.

The clustering of accessions indicated by PCA was corroborated by an NJ tree constructed from the unfiltered dataset of 1,172,469 SNPs for the 158 *T*. *turgidum* accessions (Fig 2). The wild emmers, cultivated emmers and naked wheats were again largely separated, and among the wild accessions the south emmers formed a distinct grouping, with the exception of the two *judaicum* accessions which were placed in a small clade with accessions PI 414718 and PI 467004. The tree also emphasised the distinctiveness of the Karaca Dağ emmers, and placed the three *carthlicum* accessions at the edge of the naked wheat cluster.

**Fig 3:**
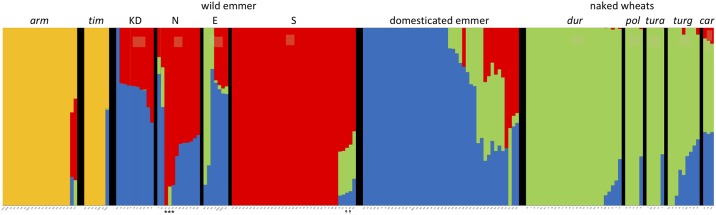
*STRUCTURE* analysis of 186 accessions based on 29,674 SNPs at *K* = 4. Each accession is shown as a vertical line divided into coloured sections, with the length of each section proportional to the membership coefficient (*Q*) of the individual accession to each of the model populations. Abbreviations: *arm*, *T*. *timopheevii* subsp. *armeniacum*; *car*, *T*. *turgidum* subsp. *carthlicum*; *dur*, *T*. *turgidum* subsp. *durum*; *pol*, *T*. *turgidum* subsp. *polonicum*; *tim*, *T*. *timopheevii* subsp. *timopheevii*; *tura*, *T*. *turgidum* subsp. *turanicum*; *turg*, *T*. *turgidum* subsp. *turgidum*; E, eastern wild emmer; KD, Karaca Dağ wild emmer; N, northern wild emmer, S, south wild emmer. The three accessions from northern Syria (PI 487263, PI 487264 and K62358) that were subsequently reassigned from the north to south wild emmer groups are indicated by asterisks, and the two *judaicum* accessions are indicated by arrows.

To reduce the computation load, the clustering algorithm *STRUCTURE* was run with a filtered dataset of 29,674 SNPs with no indels, minor allele frequency ≥0.1, minimum depth coverage of 5× and no more than 20% missing data. Using *K* = 4 as the most probable value for model complexity (based on the *ΔK* measure [52]–see S4 Fig), *T*. *timopheevii* and *T*. *turgidum* were separated with little indication of admixture between the two species (Fig 3). The only exceptions were the three *T*. *timopheevii* accessions (PI 251018, PI 427354, PI 656871) that occupied intermediate positions in the PCA and which were shown by *STRUCTURE* to be admixed with various emmer groups. Among the emmers, the wild accessions from the southern Levant displayed the least admixture, whereas the wild emmers from the three other regions were admixed with both the south wild emmers and the domesticated emmers. The naked wheat subspecies had distinctive features of their own but several accessions, especially of *T*. *turgidum* subsp. *turgidum* and *T*. *turgidum* subsp. *carthlicum* were admixed with domesticated emmer and, to a lesser extent, the south wild emmer. When the *Q*-matrix for each *T*. *turgidum* accession is displayed on a geographical map, an admixture cline within the wild emmers becomes apparent (S5A Fig), with a greater degree of admixture moving clockwise around the Fertile Crescent. The maps for domesticated emmer (S5B Fig) and the naked wheats (S5C Fig) showed that those accessions with a greater degree of admixture were largely located outside of the Fertile Crescent.

### Allele sharing

**Fig 4:**
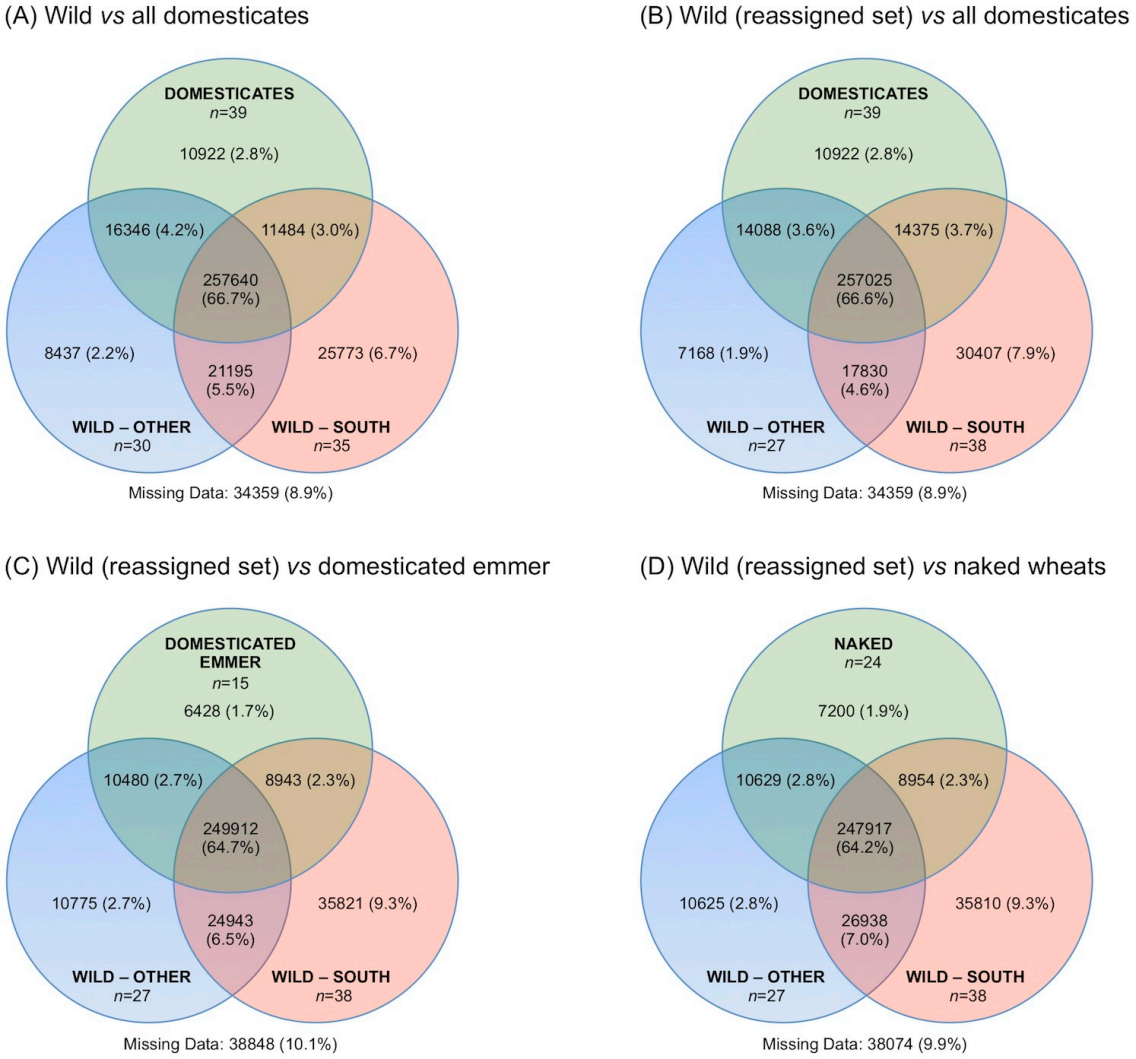
Venn diagrams showing allele sharing between different groups of wild and domesticated *T*. *turgidum* accessions. The domesticated set includes all emmer and naked wheat accessions from outside of southwest Asia (see S1 Table). (A) Analysis of all wild emmers based on 106,128 SNPs. (B) Re-analysis after transfer to accessions PI 487263, PI 487264 and K62358 from the ‘other’ to the south group. (C) Allele sharing between wild and domesticated emmers. (D) Allele sharing between wild emmers and naked wheats.

To investigate further the relationships between the different groups of wild emmer and between the wild and domesticated *T*. *turgidum* accessions we identified alleles that were private to one group or shared between groups. This analysis was carried out with a dataset of 193,077 SNPs, corresponding to 386,144 alleles, based on no indels, minimum depth coverage of 5× and no more than 20% missing data (i.e. the parameters used for *STRUCTURE* except that there was no filter for minor allele frequency). To avoid the effect of recent introgression between the wild and domesticated populations we included only those domesticated emmers and naked wheats from outside of southwest Asia (see S1 Table). Also, we combined the wild emmers from the north, Karaca Dağ and east regions into a single group, which we designated ‘other’, the justification being that the PCA, NJ and *STRUCTURE* analyses described above indicated that, whereas the south wild emmers form a relatively homogeneous population, those wild emmers from the north, Karaca Dağ and east regions are more admixed (see Figs 1–3). The allele sharing analysis showed that the majority of alleles (66.7% of the total number) were present in all three groups–south wild emmers, ‘other’ wild emmers, and domesticates (Fig 4A). A much smaller number of alleles (2.8%) were unique to the domesticated population and 3.0% and 4.2% were shared between the domesticates and either the south or ‘other’ wild groups, respectively. The analysis was repeated but with three wild emmer accessions–PI 487263, PI 487264 and K62358, collected from adjacent locations in northern Syria–moved from the ‘other’ to south grouping, the justification for this reassignment being that the *STRUCTURE Q*-matrices for these accessions were more typical of south wild emmer (see Fig 3). Following these reassignments, 3.7% and 3.6% of the alleles were shared between the domesticates and either the south or ‘other’ wild groups, respectively (Fig 4B). Similar results were obtained when allele sharing was examined for the domesticated emmers and naked wheats separately (Fig 4C and 4D).

The comparison between the reassigned wild emmer sets and domesticated emmer (Fig 4C) revealed 6428 alleles that were unique to domesticates. Most of these 6428 alleles had a relatively low frequency in domesticated emmers (mean 0.096, standard deviation 0.684) but 47 had a frequency of >0.4 (S6 Table). There were also 10,480 alleles that were present in domesticated emmer and the ‘other’ wild population but absent in south wild emmer. Although these alleles had different frequencies in the domesticated and ‘other’ wild groups, the frequency distributions were similar in the two populations (S6A Fig). Similar results were obtained when the frequency distributions of the 8943 alleles present in domesticates and south wild emmer but absent from the ‘other’ wild population were examined (S6B Fig). Analysis of the 249,912 alleles that were present in all three populations (domesticated emmer and both the south and ‘other’ wild groups) revealed that 24,799 of these alleles were fixed in the domesticates but not fixed in either of the wild populations. For each of these alleles, a frequency differential was calculated as *f*(south wild emmer) − *f*(‘other’ wild emmer) and these differentials plotted as a histogram (S7 Fig). This analysis showed that the south and ‘other’ wild populations contributed equally to those alleles that had become fixed in domesticated emmer.

To investigate whether alleles shared exclusively between the domesticated emmer accessions and either of the two wild populations were clustered in particular regions of the genome, linkage groups were constructed from the SNP data for each chromosome (S8 Fig). There was no obvious clustering: each of the chromosomes contained a mixture of SNPs shared between the domesticated emmers and either the south or other wild emmer groups (S7 Table).

### GWAS

**Fig 5:**
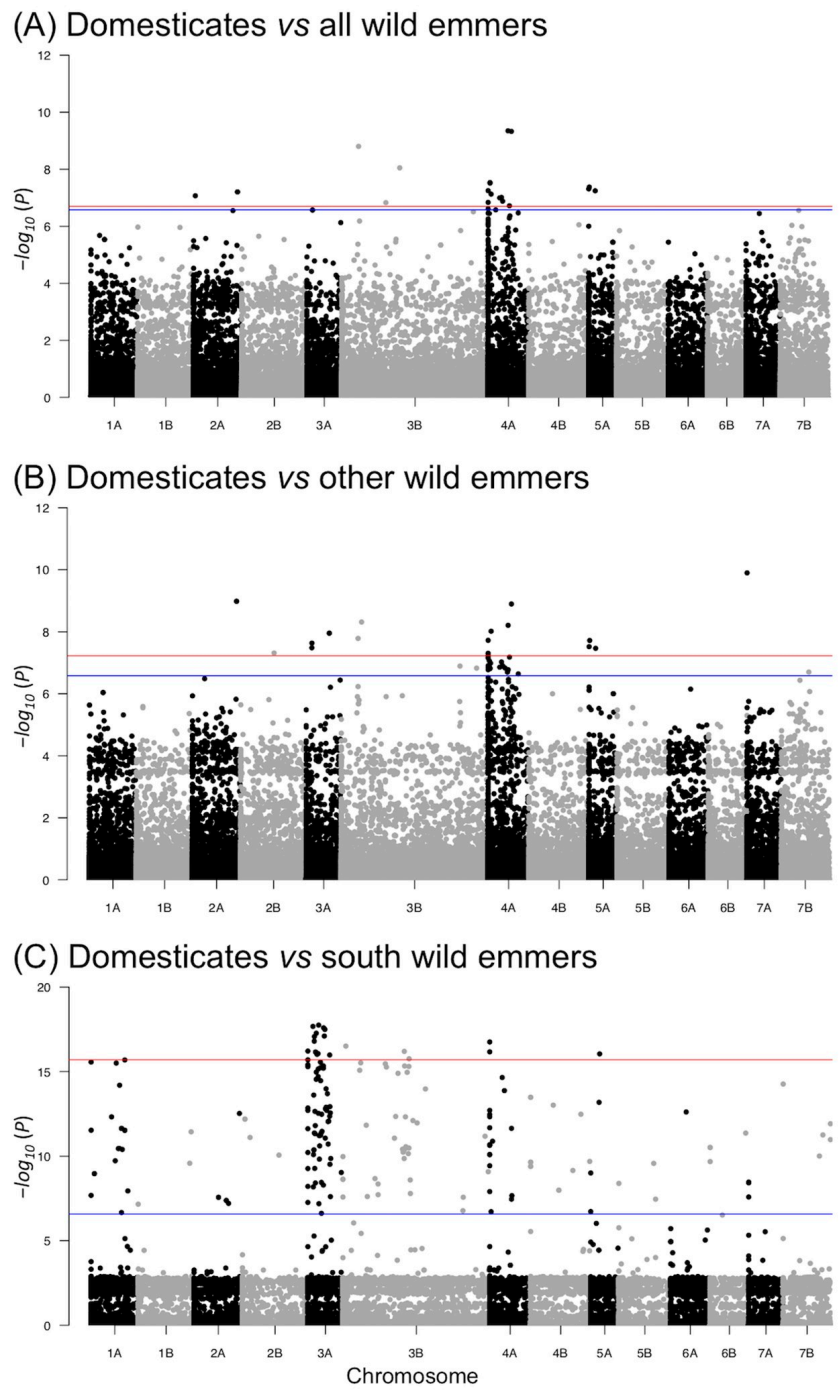
Manhattan plots displaying the results of GWAS. The analyses compare the domesticated accessions against (A) all the wild emmer accessions, (B) the ‘other’ wild emmer accessions, and (C) the south wild emmer accessions. The horizontal blue lines represent the Bonferroni threshold [*−log*_*10*_ (*P*) >6.58] and the red lines represents the 20 markers with the highest–*log*_*10*_*(P)* values.

A GWAS was performed to identify SNPs associated with the domesticated phenotype. Our approach was identical to the use of GWAS to identify markers associated with individual domestication traits such as plant architecture and seed yield [53–55]. However, we treated ‘domestication’ as a complex trait with two alternative phenotypes, domesticated and wild, in order to identify the SNPs most closely associated with the domesticated phenotype in the different populations of cultivated emmer. GWAS of the 158 *T*. *turgidum* accessions was therefore carried out with the 193,077 SNPs used in the allele sharing analysis at a *p* threshold of 6.58 × 10^−8^ (significance level of 5% after Bonferroni multiple test correction with α = 0.01 and k = 193,077 [42]). Comparison between the domesticated *T*. *turgidum* accessions and all the wild emmers revealed 22 SNPs with significance above this threshold (Fig 5A), the majority of these located on chromosome 4A. The comparison between domesticates and the ‘other’ wild accessions gave similar results, with 42 SNPs above the threshold, on eight chromosomes with the majority on 4A (Fig 5B). However, when the association analysis was carried out with the south wild accessions, substantial differences were seen. This analysis detected 196 SNPs above the Bonferroni correction threshold, these SNPs present on each of the 14 chromosomes, with the highest numbers on chromosomes 3A and 3B (Fig 5C). The significance scores for the SNPs identified in the domesticates *vs* south wild GWAS were higher than those from the other two analyses, with a maximum of *p* = 17.75 × 10^−8^, compared with 9.35 × 10^−8^ for the domesticates *vs* all analysis and 9.90 × 10^−8^ for domesticates *vs* other (S8 Table). Of the 196 significant SNPs from the domesticates *vs* south GWAS, 132 had a significance score greater than the highest scores for the domesticates *vs* all and domesticates *vs* other analyses.

Of the 22 SNPs with significance above the threshold when the wild and domesticated population were compared, 16 were also placed above the threshold in the domesticated *vs* ‘other’ comparison, one was placed above the threshold in the domesticated *vs* south comparison, and an additional SNP was above the threshold in both the ‘other’ and south comparisons (S8 Table). In each analysis, the major alleles for the SNPs above the significance threshold had a high average frequency in the domesticated population (S9 Table). In the domesticated *vs* all wild and domesticated *vs* ‘other’ wild analyses, the average frequency of these alleles was similar in both the ‘other’ and south emmer populations, However, the frequencies were significantly skewed among the significant SNPs from the domesticated *vs* south wild emmer GWAS. In this analysis the major alleles (as defined from the domesticated population) had high frequencies (mean 0.88) in the ‘other’ wild emmers, but much lower frequencies (mean 0.14) in the south wild emmers.

## Discussion

### Population structure among wild and domesticated tetraploid wheats

We used GBS with 189 tetraploid wheat accessions and obtained almost seven million tags from which we identified 1,172,469 SNPs with read depth ≥3. PCAs with SNP datasets filtered for different levels of coverage and missing data separated the *T*. *turgidum* and *T*. *timopheevii* accessions, and within *T*. *turgidum* separated the wild emmers, domesticated emmers and the naked wheats (Fig 1, S3 Fig). A few accessions occupied anomalous positions within the PCA but the majority of these anomalies were due to misclassification. In particular, ten accessions described by the germplasm collections as *T*. *turgidum* subsp. *dicoccoides* were reclassified by us as *T*. *timopheevii* subsp. *armeniacum* based on their positions in the PCA and by sequencing of the *Ppd-1* locus, which contains SNPs that enable *Ppd-B1* of *T*. *turgidum* to be distinguished from *Ppd-G1* of *T*. *timopheevii* [48]. An additional accession described as *T*. *timopheevii* subsp. *timopheevii* was reclassified by us as *T*. *turgidum* subsp. *dicoccum* on the same grounds. The original misclassification of these accessions reflects the overlapping geographic ranges and close morphological similarities of *T*. *turgidum* and *T*. *timopheevii*, with taxonomic identification based mainly on the greater degree of hairiness of the culm internodes and leaf sheaths of *T*. *timopheevii* [56]. Occasional classification errors can therefore occur.

Three accessions occupied positions in the PCAs intermediate between the *T*. *turgidum* and *T*. *timopheevii* clusters, and the identities of these accessions could not be resolved by further typing of nuclear and cytoplasmic markers (S5 Table). From the pattern of SNPs at the *pinb-A* locus [57] we tentatively identified one of these accessions (PI 427354) as an example of the wild A^m^A^m^A^m^A^m^GG hexaploid *T*. *zhukovskyi*, which again has a similar taxonomy to *T*. *timopheevii* and is easily confused with this species. Two other accessions (PI 656871 and PI 251018) gave positive results for both B and G nuclear sequences. Crosses between *T*. *turgidum* and *T*. *timopheevii* have been reported to yield F_1_ progeny (e.g. [58]), but stable hybrid lines have not been obtained and it is generally assumed that the F_1_ plants are non-infertile due to failures in chromosome pairing [59]. However, cytogenetic examination has suggested that genes can be transferred between *T*. *turgidum* and *T*. *timopheevii* by direct crosses and recombination [60], raising the possibility that PI 656871 and PI 251018 are genuine hybrids between the two species.

The PCAs also emphasized the unique features of two *T*. *turgidum* subsp. *dicoccoides* accessions belonging to the *judaicum* race, these two accessions forming a distinct cluster, along with two other wild emmers from the same geographical region close to the Sea of Galilee (Fig 1B). Members of the *judaicum* race have morphological and karyotypic features that distinguish them from other emmers [50,51], and at one time were thought to have arisen from hybridization between wild emmer and a domesticated wheat such as *T*. *turgidum* subsp. *durum* [61], though this hypothesis has been rejected by Badaeva *et al*. [51] based on a detailed study of chromosome banding patterns. *STRUCTURE* analysis of our GBS data suggests that *judaicum* has some similarity with the naked wheats (Fig 3) but this similarity was not sufficient to align these accessions with the naked wheats in the NJ tree (Fig 2).

The PCA, NJ and *STRUCTURE* analyses did not distinguish between the naked tetraploid subspecies *durum*, *polonicum*, *turgidum* and *turanicum*, in agreement with previous studies that have suggested that there is little genetic differentiation between these types [2–4]. Among the naked wheats, only subsp, *carthlicum* appears to be distinct in any way, these three accessions occupying an outlying position in the PCA of the naked wheats (Fig 1C) and forming their own cluster in the NJ tree (Fig 2), in agreement with a previous study based on analysis of simple sequence repeats (SSR) and diversity array technology (DaRT) markers, which also concluded that subsp, *carthlicum* has different genetic features compared with the other naked tetraploid wheats [2]. *Triticum turgidum* subsp. *carthlicum* has morphological similarities with *T*. *aestivum* and was initially thought to be a hexaploid wheat [62] until chromosome counts showed it to be tetraploid. It was later suggested that subsp. *carthlicum* arose from hybridization between emmer and bread wheat [63]. Our *STRUCTURE* analysis indicated that subsp. *carthlicum* is more admixed with wild and domesticated emmer than either of the other naked tetraploid subspecies (Fig 3), again in agreement with the results of SSR and DaRT analysis [2], and consistent with a model in which introgression from emmer forms part of the evolutionary history of this subspecies.

### Origins of the domesticated tetraploid wheats

The origins of the domesticated tetraploid wheats remain uncertain, the archaeological record suggesting that cultivation began in the southern Levant [11–14] and genetic studies favouring the northern regions of the Fertile Crescent [12,20,22–24]. An initial examination of the GBS data appears to lend support to the results of previous genetic studies by suggesting a relationship between domesticated *T*. *turgidum* and wild emmers from the northern Fertile Crescent. The PCA of *T*. *turgidum* accessions (Fig 1B) separates the wild emmers of the southern Levant from those obtained from the other regions of the Fertile Crescent, and places the domesticated emmers closest to these ‘other’ wild emmers. In the NJ tree (Fig 2), domesticated emmer affiliates most closely with wild *T*. *turgidum* from the Karaca Dağ region, with the southern wild emmers placed some distance away.

It has been suggested that the origins of cultivated wheat, and other crops, can be difficult to interpret from modern genetic data due to the assumptions made during certain types of analysis that the evolutionary pattern since domestication has been treelike. These assumptions have been questioned by modelling studies [27,28,64] and by analyses of genomic data which have emphasised the multiregional contribution of the wild species to the genomes of domesticated wheat [26] and barley [29,30]. In order to investigate the origin of the domesticated tetraploid wheats without making *a priori* assumptions about the evolutionary process, we examined the patterns of allele sharing between domesticated wheats and the south and ‘other’ wild populations (Fig 4). The results revealed a complex relationship between the crop and the two wild populations, and force a reappraisal of the relatively straightforward interpretation that domesticated emmer is derived solely from a wild population in the northern Fertile Crescent. We found that a significant number of alleles are shared between domesticated emmer and either, but not both, of the south or ‘other’ wild populations. The number of these shared alleles is similar in both cases, and their frequency distributions in the domesticates and the corresponding wild population are also similar (S6 Fig). The domesticated population has therefore obtained different sets of alleles from the south and ‘other’ wild emmers, each set comprising a similar number of alleles displaying a similar range of frequencies. An equivalence in the relationships between domesticated emmer and the two wild populations is also revealed when the pattern of allele sharing is examined in a second way. Those alleles that are present in all three groups (domesticates, south wild, and ‘other’ wild) include 24,799 alleles that are fixed in domesticates but not fixed in either of the wild populations. This allele set presumably includes many that have undergone selection in domesticates and which might therefore be termed ‘domestication alleles’. These alleles have different frequencies in the south and ‘other’ wild populations, but the number of these alleles that are more frequent in the south wild population is similar to the number that are more frequent in the ‘other’ wild population, and the frequency distributions in the two wild populations are similar (S7 Fig). Again, the implication is that the south and ‘other’ wild populations have contributed equally to the domesticated genepool, in this case with reference to the particular allele set that is most important in conferring the domesticated phenotype.

The pattern of allele sharing revealed by the GBS data is therefore inconsistent with a model in which domesticated emmer is derived by linear descent solely from a wild emmer population in the northern Fertile Crescent. Even if the resulting domesticated genepool was subsequently modified by hybridization with wild emmer from the southern Levant, we would not expect the modern crop to display such a similarity in allele sharing with both the south and ‘other’ wild populations. By the same argument, a model in which emmer is domesticated in the southern Levant and subsequently hybridizes with wild populations from the north, can also be discounted. The most rational explanation of the allele sharing data is that domesticated emmer is derived from a mixed population that combined the genetic features of the modern southern and northern wild populations. This mixed population could have been a naturally occurring wild population, but such a population does not appear to exist at the present time; this explanation would therefore require that there has been a substantial change in the wild phylogeography over the last 10,000 years. Alternatively, the mixed population could have been an artificial pre-domesticated population created by the early cultivators, consistent with models in which emergence of the domesticated versions of wheat and barley was preceded by a lengthy period during which plants with wild phenotypes were cultivated [65–67].

Is it possible to rationalize a model in which the first domesticates are derived from a mixed population, combining the features of wild emmer from both the southern Levant and northern Fertile Crescent, with the results of the PCAs and NJ analysis which suggest that domesticated emmer has greater affinity with the northern wild population? Civáň et al. [26] have previously suggested that wild emmer was first cultivated in the southern Levant, but remained a pre-domesticated population until cultivation spread out of this core area into the northern Fertile Crescent, where the crop began to acquire the domesticated phenotype. According to this model, the wild emmers found today in the northern Fertile Crescent are feral descendants of this pre-domesticated population. Hence the origin of cultivation is the southern Levant, but domesticated emmer has closer affinities with the modern wild population in the Fertile Crescent. The results of our GBS analyses are compatible with this model if one modification is introduced. We propose, in agreement with Civáň et al. [26], that emmer was first cultivated in the southern Levant and that this pre-domesticated population then spread to the northern Fertile Crescent as agriculture expanded out of its core, southern area. However, unlike Civáň et al. [26], we suggest that wild emmer was already present in the northern Fertile Crescent, and that the cultivated, pre-domesticated emmer from the southern Levant mixed with this northern wild emmer, giving rise to the mixed population from which the first domesticated emmers emerged, in accordance with the allele sharing data. The cultivated emmer brought from the southern Levant, although still displaying a non-domesticated phenotype, had already begun to undergo selection for alleles favourable for domestication. Hence in the modern domesticates, the highest GWAS scores are obtained when domesticates are compared with this source southern wild emmer population (S8 Table). Following mixing with the northern wild emmers, the domestication traits began to appear in the cultivated population, fixation of these traits possibly aided by the scattered and non-abundant distribution of wild emmer in the northern Fertile Crescent [7], which would have promoted reproductive isolation between the crop and wild plants. As suggested by Civáň et al. [26], feralization of the component of the pre-domesticated population that did not acquire domestication traits would contribute a genetic signature to the northern wild emmer, this input resulting in the modern northern population displaying features of both the domesticates and wild emmer from the southern Levant, as shown by our *STRUCTURE* analysis (Fig 3). In less discriminatory analyses where a treelike evolutionary pattern is assumed, the complex structure of northern wild emmer could result in apparent derivation of the crop solely from this population, as suggested by our NJ tree (Fig 2) and by previously reported analyses based on tree-building.

## Conclusions

We show that SNP typing by GBS is capable of providing robust information on the genetic relationships between species and subspecies of wild and cultivated tetraploid wheat. For species without large genomes, for which complete genome resequencing is currently impractical, GBS provides a rapid means of acquiring diversity data from all parts of the genome. The wealth of data provided by GBS enables relationships between different groups of tetraploid wheats to be examined in greater detail than has previously been possible. This analysis reveals that the evolutionary origins of domesticated tetraploid wheat are complex, which has been suggested by previous studies (e.g. [26]) and is in accordance with the equivalent complexity being revealed by studies of other crops such as barley [29,30] and rice [31].

## Supporting information

S1 Fig Collection sites for wild emmer accessions. The annotations show the division into south (S), north (N), Karaca Dağ (KD) and east (E) groups. Map drawn using ArcMap v.10 of ArcGIS [42].
(TIFF)

S2 Fig Relationship between number of mapped SNPs and chromosome length. Chromosomes from the A genomes are shown in red, B in blue, and D in orange. Regression lines are shown with closeness of fit indicated by the r^2^ values given at the bottom right of the panel.
(TIFF)

S3 Fig PCAs of 186 *T*. *turgidum* and *T*. *timopheevii* accessions with different filters for missing data and coverage. (TIFF)

S4 Fig Identification of the most probable value for model complexity. *K* is plotted against *ΔK* in accordance with ref [51]. The peak at *K* = 4 indicates that this is the most probable value, excluding the peak at *K* = 2 which simply separates *T*. *turgidum* and *T*. *timopheevii*.
(TIFF)

S5 Fig Geographical distribution of the population structure for the *T*. *turgidum* accessions. Each pie chart is an individual accession coloured according to the *Q*-matrix at *K* = 4. (A) Wild emmer accessions. (B) Domesticated emmer accessions. (C) Naked wheats. In panel C, The *T*. *turgidum* subsp. *durum* accession PI 61164 from Russia is absent because it is located off of this map. This accession belongs entirely to the ‘green’ population. Maps drawn using ArcMap v.10 of ArcGIS [42].
(TIFF)

S6 Fig Frequency distributions for alleles shared between domesticated emmers and (A) the ‘other’ wild group, and (B) south wild emmers. (TIFF)

S7 Fig Frequency differentials for 24,799 alleles that are fixed in domesticated emmers but not fixed in the south or ‘other’ wild populations. Frequency differentials were calculated from *f*(south wild emmer) − *f*(‘other’ wild emmer). The left side of the histogram shows those alleles that are more frequent in the ‘other’ wild emmer population, and the right side shows those alleles more common in the south emmers. The histogram is drawn with bin sizes of 100 alleles.
(TIFF)

S8 Fig Linkage groups constructed from the allele sharing data displayed in Fig 4B. The positions of SNPs with one or both alleles shared between domesticated emmer and the south wild emmers are shown in red, and those shared between domesticated emmer and the other wild emmers are shown in green. The positions of all other SNPs are shown in grey.
(TIFF)

S1 Table Wheat accessions used in this study. (XLSX)

S2 Table GBS data. (XLSX)

S3 Table Data for mapped SNPs. (XLSX)

S4 Table SNP summary statistics for individual taxa. (XLSX)

S5 Table Reclassification of misidentified accessions. (XLSX)

S6 Table Alleles unique to the domesticated emmer population and with a frequency of >0.4. (XLSX)

S7 Table Numbers of alleles per chromosome that are shared between wild emmers (reassigned set) and south or ‘other’ domesticates (data shown in Fig 4B). (XLSX)

S8 Table SNPs above the significance threshold in the three GWAS analyses. (XLSX)

S9 Table Average major allele frequencies for SNPs above the significance threshold in the three GWAS analyses. (XLSX)
