# Supplementary figures and images for: Multiregional origins of the domesticated tetraploid wheats
Source: PLoS One. 2020 Jan 22;15(1):e0227148. doi: 10.1371/journal.pone.0227148 (PMC6975532; doi:10.1371/journal.pone.0227148)

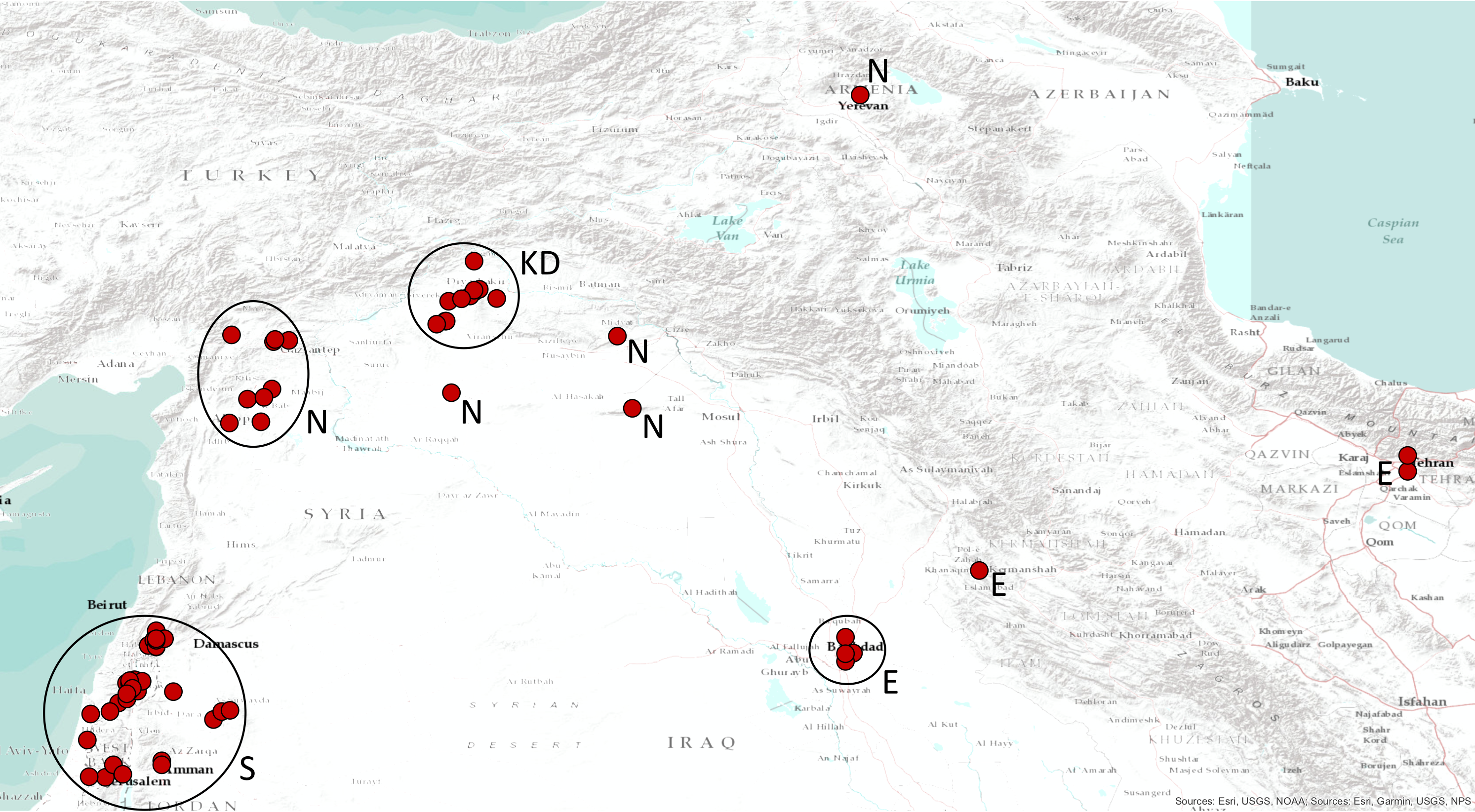

Supplement: S1 Fig — The annotations show the division into south (S), north (N), Karaca Dağ (KD) and east (E) groups. Map drawn using ArcMap v.10 of ArcGIS [42]. (TIFF) [file pone.0227148.s001.tiff]

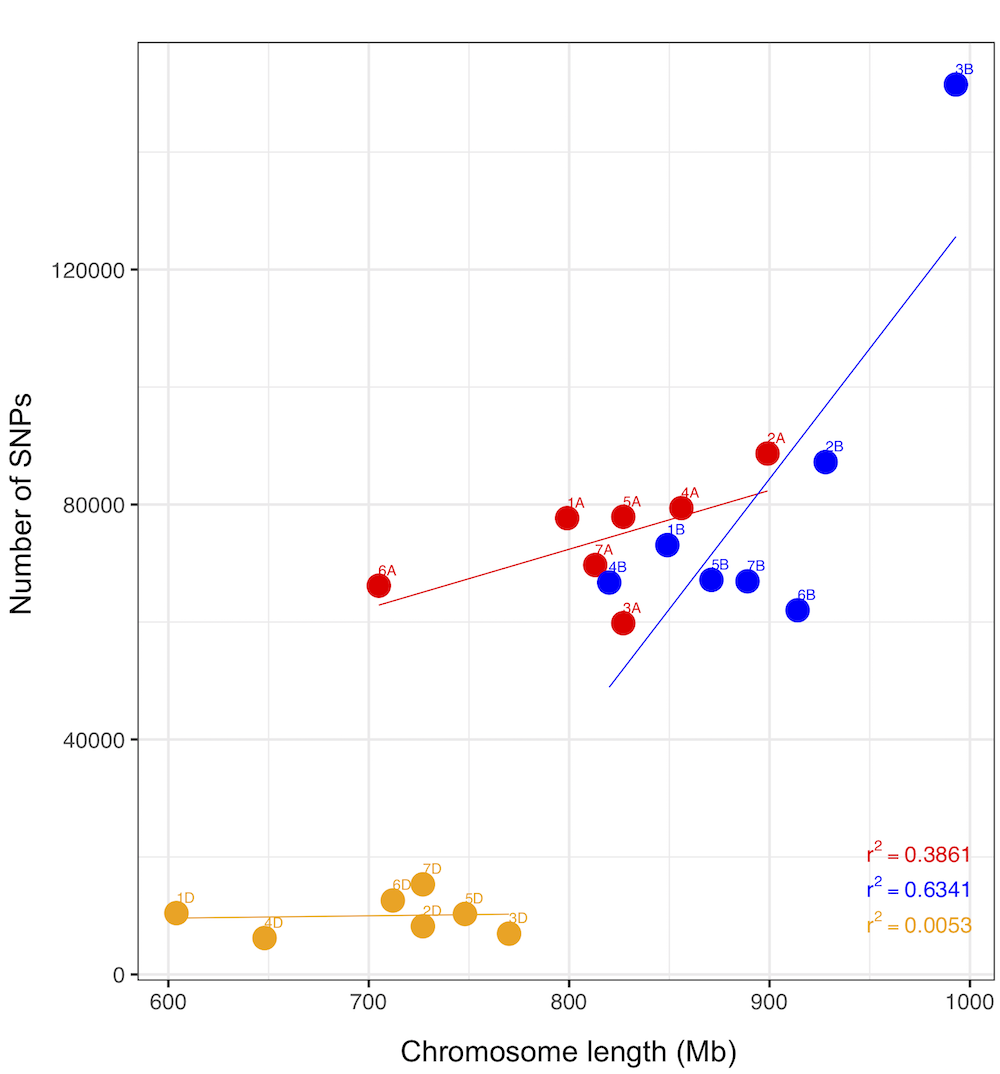

Supplement: S2 Fig — Chromosomes from the A genomes are shown in red, B in blue, and D in orange. Regression lines are shown with closeness of fit indicated by the r2 values given at the bottom right of the panel. (TIFF) [file pone.0227148.s002.tiff]

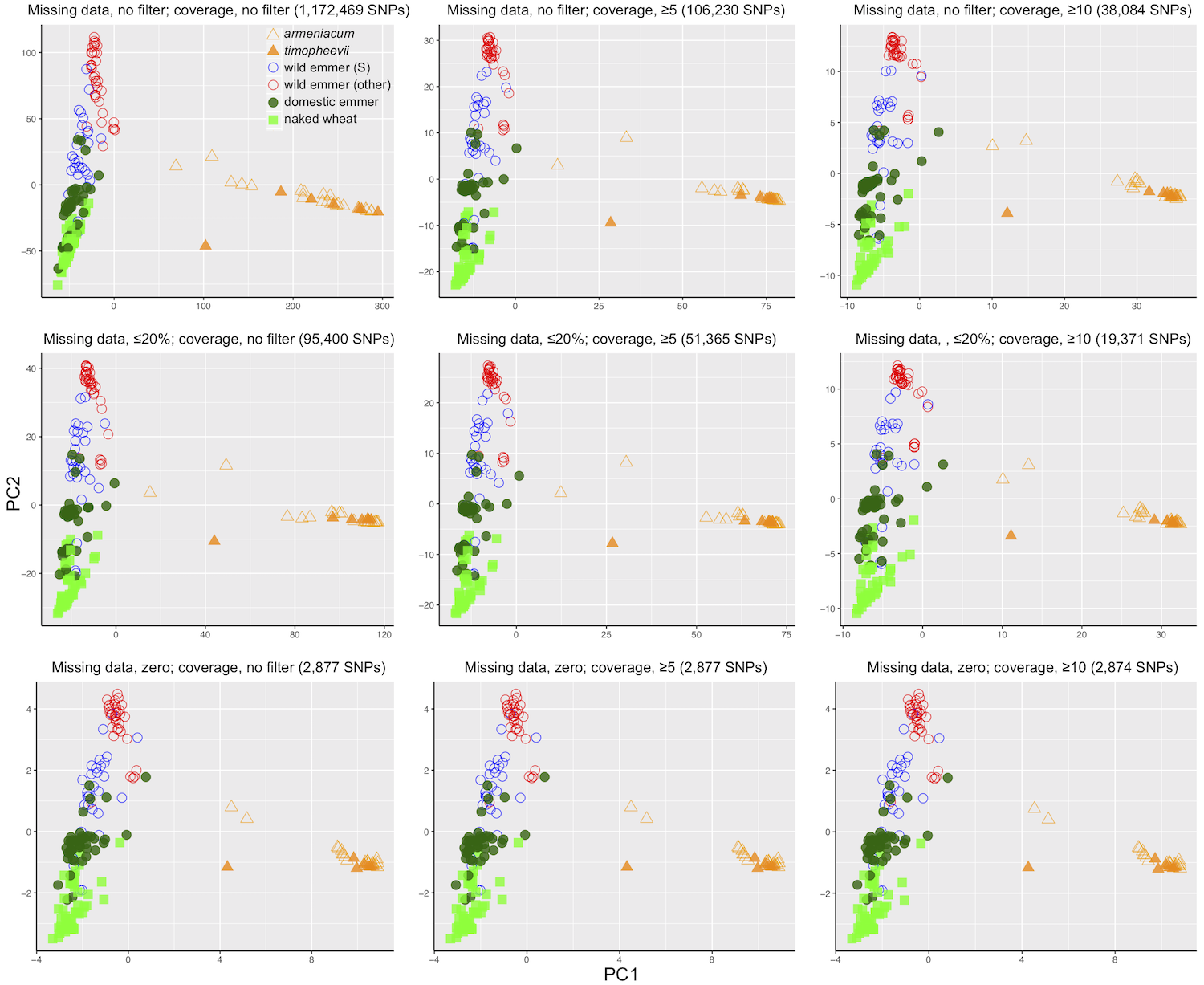

Supplement: S3 Fig — (TIFF) [file pone.0227148.s003.tiff]

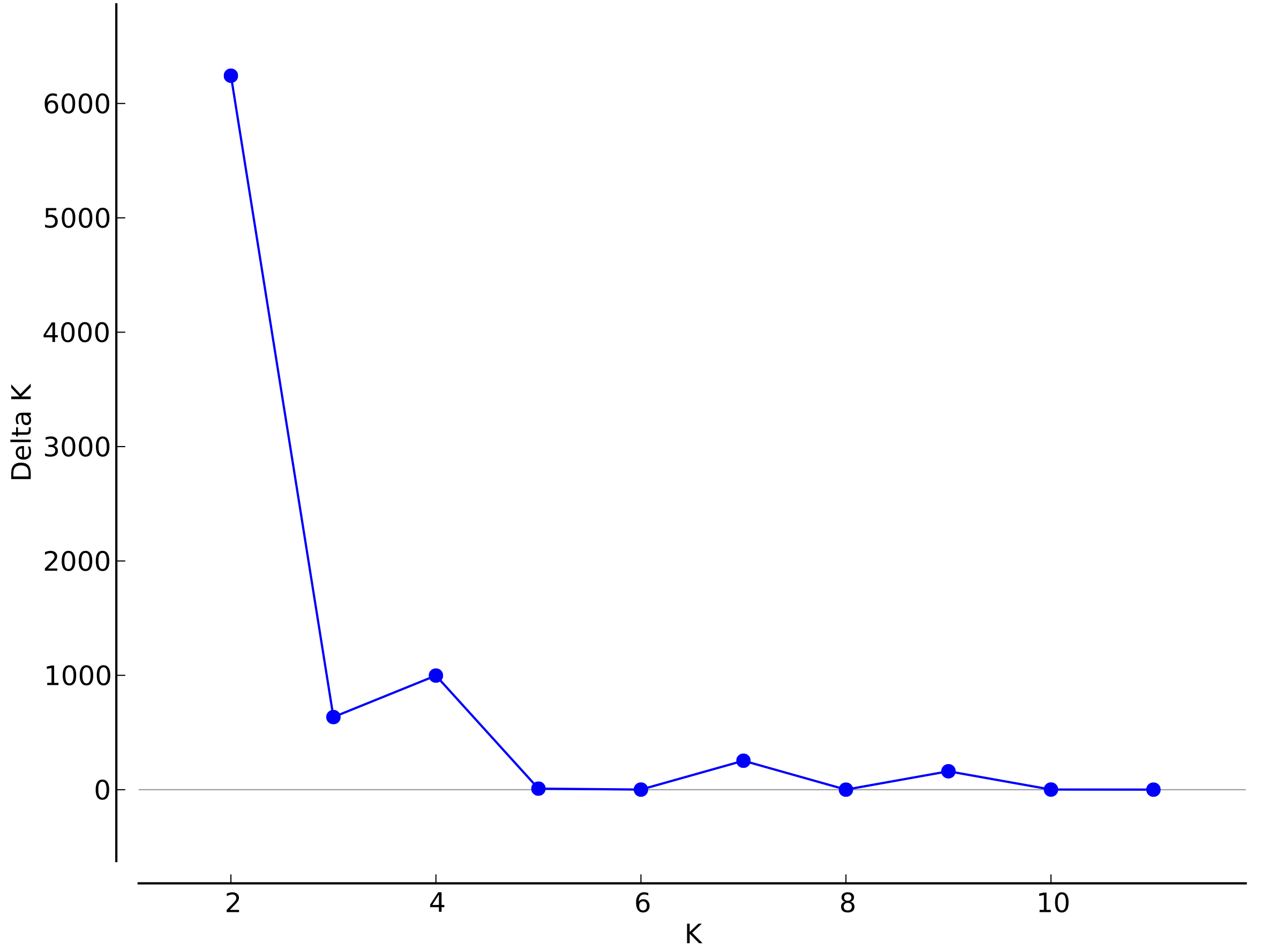

Supplement: S4 Fig — K is plotted against ΔK in accordance with ref [51]. The peak at K = 4 indicates that this is the most probable value, excluding the peak at K = 2 which simply separates T. turgidum and T. timopheevii. (TIFF) [file pone.0227148.s004.tiff]

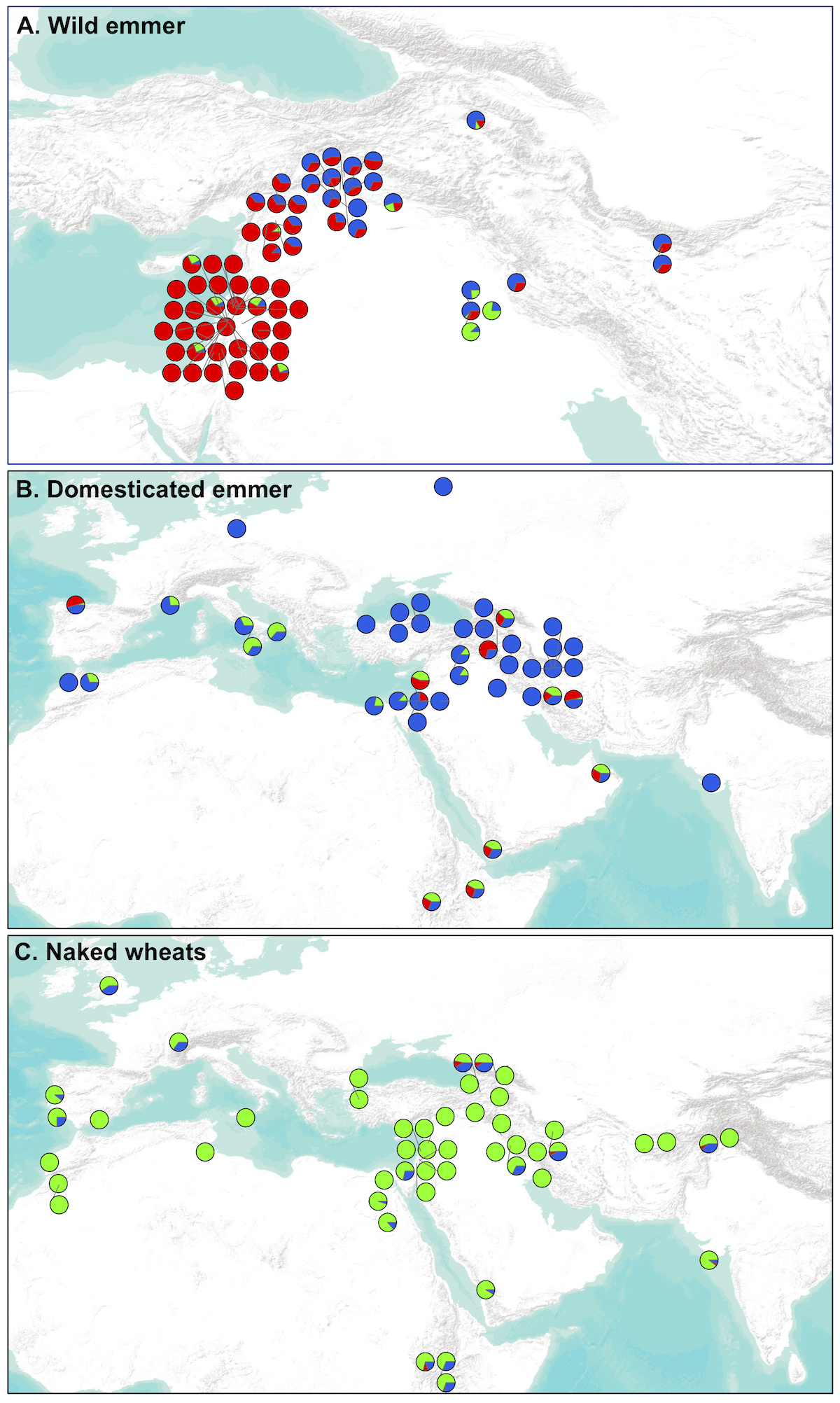

Supplement: S5 Fig — Each pie chart is an individual accession coloured according to the Q-matrix at K = 4. (A) Wild emmer accessions. (B) Domesticated emmer accessions. (C) Naked wheats. In panel C, The T. turgidum subsp. durum accession PI 61164 from Russia is absent because it is located off of this map. This accession belongs entirely to the ‘green’ population. Maps drawn using ArcMap v.10 of ArcGIS [42]. (TIFF) [file pone.0227148.s005.tiff]

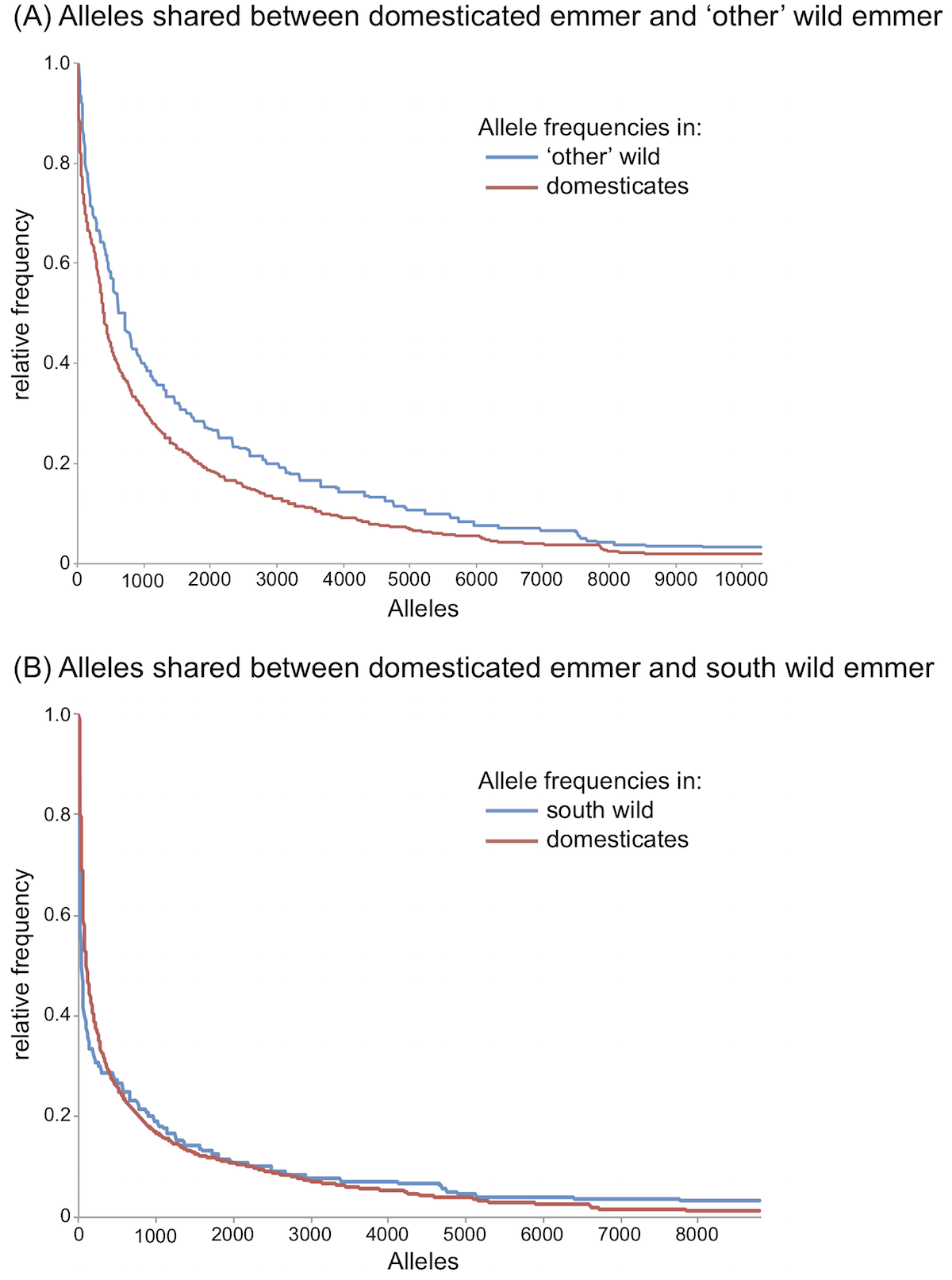

Supplement: S6 Fig — (TIFF) [file pone.0227148.s006.tiff]

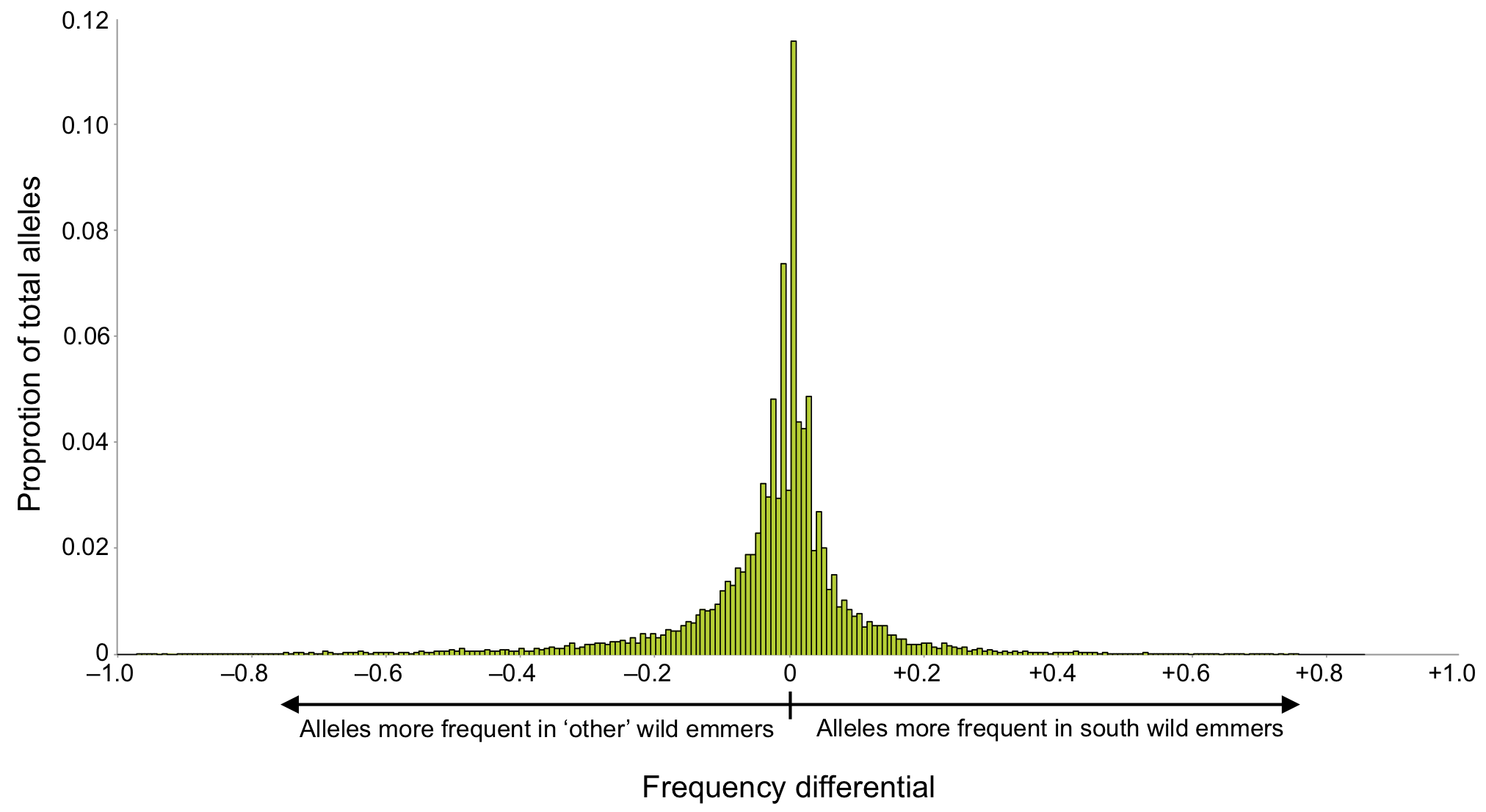

Supplement: S7 Fig — Frequency differentials were calculated from f(south wild emmer) − f(‘other’ wild emmer). The left side of the histogram shows those alleles that are more frequent in the ‘other’ wild emmer population, and the right side shows those alleles more common in the south emmers. The histogram is drawn with bin sizes of 100 alleles. (TIFF) [file pone.0227148.s007.tiff]

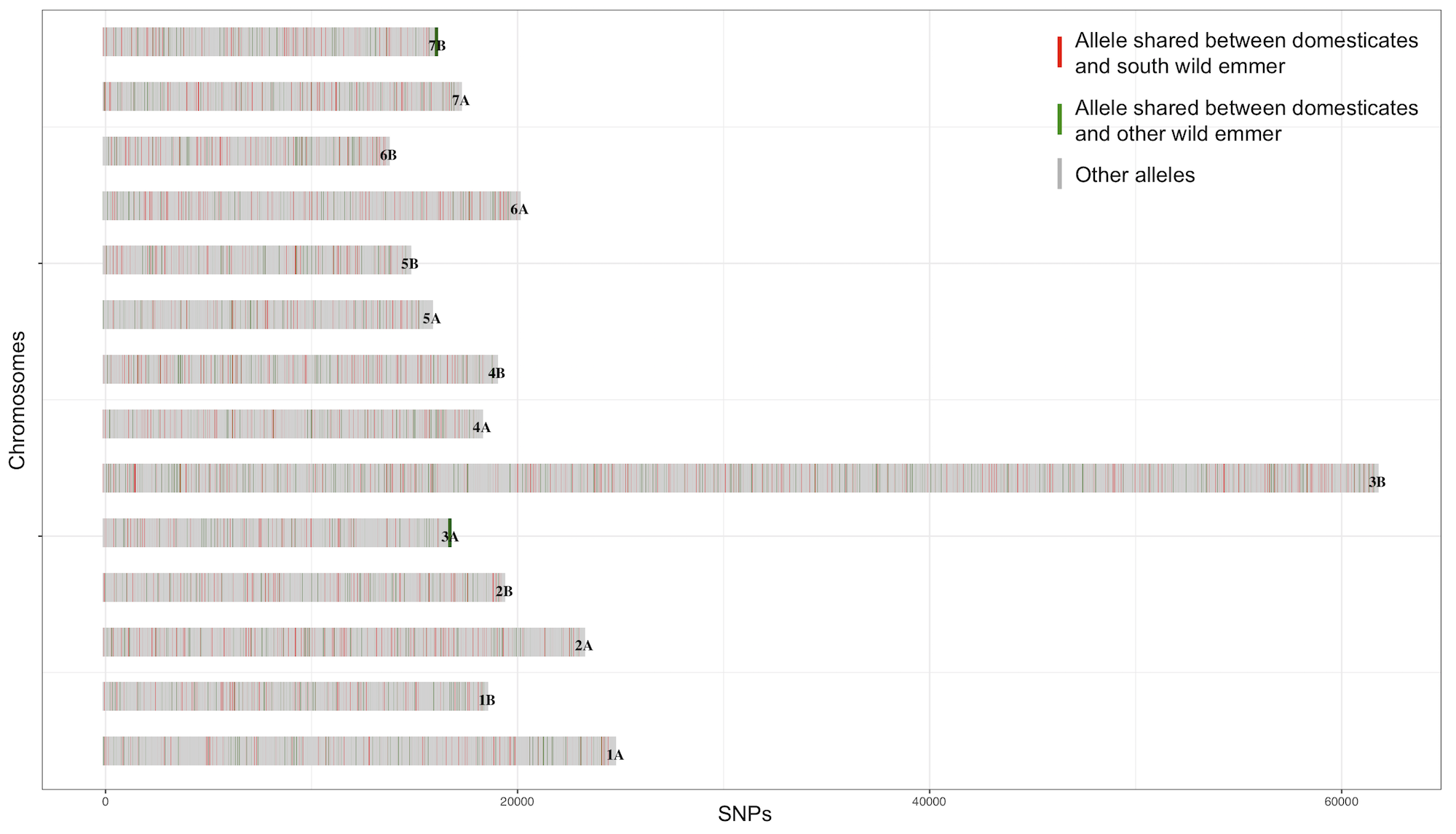

Supplement: S8 Fig — The positions of SNPs with one or both alleles shared between domesticated emmer and the south wild emmers are shown in red, and those shared between domesticated emmer and the other wild emmers are shown in green. The positions of all other SNPs are shown in grey. (TIFF) [file pone.0227148.s008.tiff]
